# Association between Periodontitis and High Blood Pressure: Results from the Study of Periodontal Health in Almada-Seixal (SoPHiAS)

**DOI:** 10.3390/jcm9051585

**Published:** 2020-05-23

**Authors:** Vanessa Machado, Eva Muñoz Aguilera, João Botelho, Syed Basit Hussain, Yago Leira, Luís Proença, Francesco D’Aiuto, José João Mendes

**Affiliations:** 1 Periodontology Department, Clinical Research Unit (CRU), Centro de Investigação Interdisciplinar Egas Moniz (CiiEM), Instituto Universitário Egas Moniz (IUEM), 2829-511 Caparica, Portugal; jbotelho@egasmoniz.edu.pt; 2 Clinical Research Unit (CRU), Centro de Investigação Interdisciplinar Egas Moniz (CiiEM), Instituto Universitário Egas Moniz (IUEM), 2829-511 Caparica, Portugal; jmendes@egasmoniz.edu.pt; 3 Periodontology Unit, University College London Eastman Dental Institute, 21 University Street, London WC1E 6DE, UK; eva.aguilera.15@ucl.ac.uk (E.M.A.); syed.hussain.16@ucl.ac.uk (S.B.H.); y.leira@ucl.ac.uk (Y.L.); f.daiuto@ucl.ac.uk (F.D.); 4 Department of Periodontology, Faculty of Dentistry, Universitat Internacional de Catalonia, 08195 Barcelona, Spain; 5 Medical-Surgical Research Group, Health Research Institute of Santiago de Compostela, 15706 Santiago de Compostela, Spain; 6 Quantitative Methods for Health Research (MQIS), Centro de Investigação Interdisciplinar Egas Moniz (CiiEM), Instituto Universitário Egas Moniz (IUEM), 2829-511 Caparica, Portugal; lproenca@egasmoniz.edu.pt

**Keywords:** hypertension, blood pressure, systolic blood pressure, diastolic blood pressure, periodontitis, periodontal diseases

## Abstract

Periodontitis is a common chronic inflammatory disease which could have an important impact on blood pressure (BP). This study aimed to explore (a) the association between periodontal health and BP in a large representative cohort, (b) the predictive value of diagnosis of periodontitis in undiagnosed raised BP and (c) whether age is a mediator of this relationship. In total, 1057 randomly recruited individuals (mean age, 60.9 ± 16.3 years, 57.7% women) underwent periodontal clinical assessment and one-single BP measurement using an automated sphygmomanometer device. Logistic and linear regression models were used to estimate the odds of hypertension based on periodontitis case definitions. Mediation analysis was performed to understand the effect of age on the association of periodontitis with hypertension. Adjusted logistic model for gender, smoking habits and body mass index confirmed the association between high BP and periodontitis (OR = 2.31, 95%CI: 1.75–3.04, *p* < 0.001). Among 168 participants with undiagnosed high BP (15.9% of the study sample), 62.5% had periodontitis (*n* = 105). In this study, the association between periodontitis with both systolic blood pressure (SBP) (77.6%, *p* < 0.001) and diastolic blood pressure (DBP) (66.0%, *p* < 0.001) was mediated by age. Periodontitis is closely linked to BP in a representative Portuguese population.

## 1. Introduction

Hypertension is one of the most prevalent cardiovascular diseases (CVD), affecting 1.3 millions of people worldwide [1,2,3,4,5]. It is related to cardiovascular complications, increased morbidity, mortality and costs for society [3,6,7,8]. Hypertension has a complex aetiology, with more evidence suggesting an increased role of inflammation in the development of this condition [9]. It is a primary modifiable risk factor for cardiovascular, cerebrovascular and renal diseases [10,11], therefore, early diagnosis and treatment of elevated blood pressure (BP) is key in preventing complications and improving the general health of the population worldwide.

Periodontitis is a chronic non-communicable inflammatory disease of the supporting tissues of the teeth, with the prevalence of its severe form in 2015 reaching 616 million worldwide [12]. The oral sequelae of the disease, that if untreated ultimately leads to tooth loss, increases morbidity, reduces quality of life and work productivity [13]. Current evidence suggests periodontitis as a risk factor for CVDs, metabolic conditions, chronic respiratory and neurological disorders, rheumatoid arthritis, immunity conditions, stress or cancer [14,15,16,17,18,19,20,21]. Similarly, epidemiological and limited interventional studies worldwide have observed an association between periodontitis and hypertension [18,22]. Periodontitis-related low-grade systemic inflammation could be at the basis of this association as already linked to vascular stiffness and dysfunction, which could, in turn, contribute to an increased systemic vascular resistance leading to hypertension [23,24,25]. Recent evidence highlighted the role of periodontal bacteria in atherosclerosis and vascular dysfunction [26,27]. The management of periodontitis may offer the opportunity for researchers and clinicians to help tackle hypertension and its complications.

A recent study from our group, including a representative sample of the population in the southern Lisbon Metropolitan Area, has shown one of the highest prevalences of periodontitis in Europe [28,29]. Almost 60% of the target population was estimated to have periodontitis, with 46% being moderate and severe cases [28]. Similarly, the prevalence of hypertension in Portugal was 36.0%, with higher estimates in males (39.6%) and among those aged 65 to 74 years (71.3%) [30], which exceeded the worldwide reported prevalence [31]. There is, however, limited evidence regarding the link between periodontitis and hypertension in Portugal. Hence, the aim of this study was to investigate the association between periodontitis and hypertension in a representative sample of the southern Lisbon Metropolitan Area population. The primary objective was to assess the risk of hypertension in participants with periodontitis, and secondary objectives were to further investigate the level of undetected/undiagnosed raised BP in participants with periodontitis, and whether age can act as a possible mediator of this relationship.

## 2. Materials and Methods

### 2.1. Study Design and Population

Study of Periodontal Health in Almada-Seixal (SoPHiAS) is a population-based cross-sectional study in the southern Lisbon Metropolitan Area, involving the cities of Almada and Seixal [28]. Patients were recruited from the 22 health centres grouping of Almada-Seixal (ACES Almada-Seixal). Eligible participants were randomly drawn, proportional to each health center population, and stratified by age, as fully detailed elsewhere [28]. A representative sample of 1064 adults 18–95 years of age gave consent to the study and was examined. Considering the risk of existing gestational hypertension [32], pregnant women (*n* = 7) were excluded, resulting in a final sample of 1057 participants. This study was approved by the Research Ethics Committee of the Regional Health Administration of Lisbon and Tagus Valley, IP (Portugal) (approval numbers: Process 3525/CES/2018 and 8696/CES/2018).

All patients gave written informed consent and completed socio-demographic and medical questionnaires, including medication inventory. Additionally, anthropometric measurements, BP measurements and an oral examination with periodontal records were collected.

### 2.2. Blood Pressure Assessment

Using an automated sphygmomanometer device (Omron M3 Comfort^®^), BP readings were carried as a one-single measure [33]. Patients avoided caffeine, exercise and smoking in the 30 min prior to BP measurement. Moreover, patients remained seated for 3–5 min without talking or moving around before recording the BP reading, and patients were relaxed, sitting in a chair with feet flat on the floor and back supported. Both the patient and the observers did not talk during the rest and measurement periods. The patient’s arm was resting on a desk, and the middle of the cuff was positioned on the patient’s upper arm at the level of the right atrium, with the bladder encircling 75%–100% of the arm. Systolic and diastolic BP (SBP and DBP) were recorded to the nearest value, and these readings were provided, both verbally and in writing, to each patient [34]. Overall average SBP, DBP and pulse were used in a continuous format. Further, hypertension was defined as values of SBP ≥ 140 mmHg or DBP ≥ 90 mmHg, or; the use of antihypertensive medication [35,36].

### 2.3. Periodontal Examination

Periodontal clinical recordings were performed by two trained and calibrated examiners, as previously described [28]. A full-mouth periodontal assessment was carried out, excluding third molars, dental implants and retained roots, using a manual periodontal probe (UNC 15 probe, Hu-Friedy, Chicago, IL, USA). The number of missing teeth was recorded. Further, dichotomous plaque index (PI) [37], gingival recession (Rec), probing pocket depth (PPD), and bleeding on probing (BoP) [38], periodontal inflamed surface area (PISA) and periodontal epithelial surface area (PESA) [39] were circumferentially recorded at six sites per tooth (mesiobuccal, buccal, distobuccal, mesiolingual, lingual, and distolingual). PPD was measured as the distance from the free gingival margin to the bottom of the pocket and Rec as the distance from the cemento-enamel junction (CEJ) to the free gingival margin, and this assessment was assigned a negative sign if the gingival margin was located coronally to the CEJ. Clinical attachment loss (CAL) was calculated as the algebraic sum of Rec and PPD measurements for each site. The measurements were rounded to the lowest whole millimeter.

Periodontal status was defined following the latest available EFP/AAP consensus for gingivitis and periodontitis cases [40,41] and used as categorical independent variables. A gingivitis case was defined if a total score of BoP ≥ 10% [41]. Periodontitis case was defined if interdental CAL is detectable at ≥2 non-adjacent teeth, or buccal or oral CAL ≥ 3 mm with PPD > 3 mm at ≥2 teeth. Periodontitis staging was defined according to severity and extent [40]. Concerning severity, interdental CAL at the site of the greatest loss of 1–2 mm, 3–4 and ≥5 was considered as mild (Stage 1), moderate (Stage 2), and severe (Stage 3 and Stage 4), respectively [40].

### 2.4. Additional Study Covariates

Additional study covariates were collected via sociodemographic and medical questionnaires. Among these covariates were gender, age, marital status (single, married/union of fact, divorced or widowed), occupation (student, employed, unemployed or retired) and smoking habits (current status: never, former, current). Education was categorized according to the 2011 International Standard Classification of Education (ISCED-2011) (UNESCO 2012): no education (ISCED 0 level), elementary (ISCED 1–2 levels), middle (ISCED 3–4 levels), higher (ISCED 5–8 levels). Comorbidities were categorized according to Aimetti et al. (2015) and diabetes mellitus (DM) was confirmed using WHO criteria [42]. Measurements of height and weight were taken at the clinical exam and body mass index (BMI) was calculated as kg/m2.

### 2.5. Statistical Analysis

Data were analyzed by means of descriptive and inferential methodologies, using IBM SPSS Statistics v.25 software. The level of statistical significance was set at 5%. Descriptive measures are reported through mean ± standard deviation (SD) for continuous variables and number of cases (*n*), percentage (%) for categorical variables. We compared baseline variables between periodontitis and non-periodontitis groups using Chi-square test for categorical variables and Mann–Whitney test for continuous variables. Multivariate analyses (binary logistic and linear regressions) were used to model the influence of the periodontal status on arterial hypertension. Logistic regression analyses were performed, accounting for periodontitis staging, for all participants, and as a function of antihypertensive medication use. Odds ratio (OR) and 95% confidence intervals (95% CI) were calculated within the logistic regression analyses, for different adjustment levels. Model variables were selected among clinical and demographic characteristics. Following the initial crude model (model 1), four progressively adjusted models were generated (model 2: gender; model 3: gender and smoking habits; model 4: gender, smoking habits and BMI; model 5: gender, smoking habits, BMI and age). Furthermore, linear regression analyses were used to construct multivariate models to evaluate the influence of continuous variables on SBP and DBP readings. Age, BMI and periodontal clinical variables, such as mean CAL, mean PDP, %PPD ≥ 3, 4, 5, 6 and 7, %CAL ≥ 3, 4, 5, 6 and 7, mean Rec, PESA, PISA, BoP and missing teeth were considered. The impact of each variable to the model was appraised through Wald statistics, within stepwise procedures. Following the initial crude models, reduced adjusted models were generated for SBP and DBP. Finally, we used a mediation model to identify and explain the other pathways or processes underlying the relationship between periodontitis (independent variable) and SBP/DBP (dependent variable), via the hypothetical mediator of age. Additionally, in order to investigate if periodontal parameters might improve prediction of hypertension diagnosis, a receiver operating characteristic (ROC) analysis, with its area under the curve (AUC), was performed (Supplemental Information, Figures S1 and S2).

## 3. Results

### 3.1. Baseline Characteristics of the Study Group

**Figure 1:**
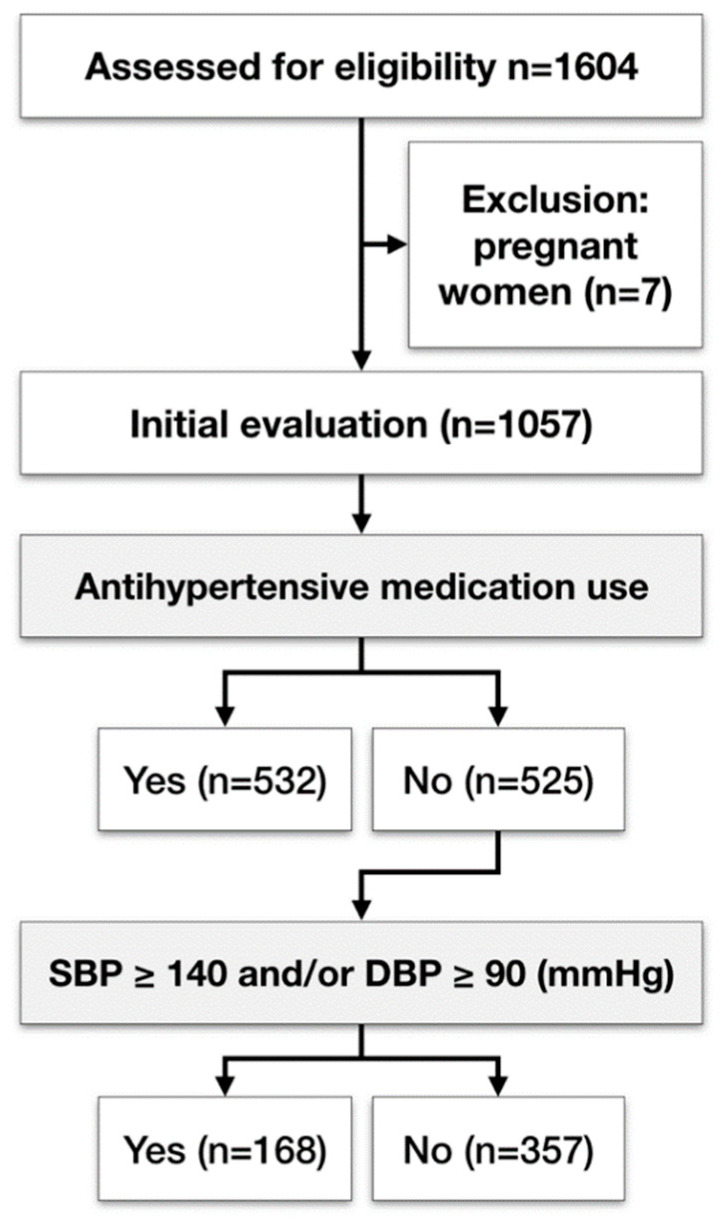
Flow diagram of cohort selection of patients from the Study of Periodontal Health in Almada-Seixal.

All participants were recruited between December 2018 and April 2019 and they were predominantly Caucasians (86.7%). The prevalence of periodontitis was 60.0% in this population subset. Those participants with periodontitis were mainly males (68.7% vs. 53.6%, *p* < 0.001), older (65.2 ± 13.5 years, *p* < 0.001), predominantly smokers (68.3%, *p* < 0.001), presenting with lower education levels (more than double figures of individuals with no or basic education, 73.8% and 67.3%, *p* < 0.001), with greater prevalence of DM (66.4%, *p* < 0.001) and hypertension (74.0%, *p* < 0.001), when compared to participants with no clinical signs of periodontitis. Indeed, the number of medical conditions recorded was almost double in the periodontitis group versus controls (*p* < 0.001) (Table 1 and Table S1). Mean ± SD of SBP and DBP measured were 136.5 ± 20.4 and 79.6 ± 13.6 mmHg in participants with periodontitis versus 129.5 ± 20.1 and 77.9 ± 13.2 mmHg for those without, respectively (*p* < 0.001 for SBP and *p* = 0.082 for DBP). More than 50% of the participants (*n* = 588) reported being diagnosed with hypertension, of which 532 participants were taking antihypertensive on the date of the study visit. Of those participants who did not take antihypertensive (*n* = 525), one hundred and sixty-eight (32%) had SBP ≥ 140 or DBP ≥ 90 (Figure 1), of which 105 (62.5%) had periodontitis. Among the patients who did not take antihypertensive and with no elevated BP (*n* = 357), 47.3% had periodontitis (*n* = 169).

### 3.2. Association Between Periodontitis and Hypertension

In the crude model (model 1), the association of all stages of periodontitis with hypertension was statistically significant (OR = 1.72, 95% CI: 1.10–2.57 for mild periodontitis; OR = 2.60, 95% CI: 1.82–3.72 for moderate periodontitis; and OR = 2.20, 95% CI: 1.57–3.08 for severe periodontitis) (Table 2). However, the fully adjusted analysis confirmed a raised, but not statistically significant, association when age was included in model 5 (OR = 1.36, 95% CI: 0.84–2.18 for mild periodontitis; OR = 1.41, 95% CI: 0.92–2.15 for moderate periodontitis; and OR: 1.04, 95% CI: 0.69–1.55 for severe periodontitis) (Table 2). In patients not taking antihypertensive medication, high BP was mostly associated with moderate periodontitis in the crude model (OR = 2.60; 95% CI: 1.61–4.21), and after adjusting for gender, smoking habits and BMI (OR = 2.60; 95% CI: 1.59–4.26).

Then, we investigated if periodontal parameters and the periodontal diagnosis could improve the prediction of undetected hypertension diagnosis with ROC analyses. We confirmed that the addition of periodontal characteristics only slightly improved the hypertension cases prediction (Appendices S1 and S2).

Linear regression models confirmed that the percentage of sites with PPD ≥ 6 mm and BoP showed a positive significant association with increased SBP and DBP (ß coefficient = 45.47, SE = 12.37, *p* < 0.001; ß coefficient = 40.73, SE = 9.93, *p* < 0.001, respectively); moreover, age, BMI, and mean CAL were also significant (Table 3).

Sensitivity analyses evidenced age and BMI as common related factors to SBP. Yet, in participants not taking antihypertensive medications, BoP was associated with SBP, whilst for those patients using antihypertensive drugs, the percentage of sites with PPD ≥ 7 mm and the number of missing teeth were closely linked to SBP. Similarly, age and BMI were the strongest predictors of DBP in participants not taking anti-hypertensives, whilst PPD ≥ 6 and BoP remained associated with DBP in those taking BP medications (Table 3).

### 3.3. Age Effect on the Periodontitis–Hypertension Link

**Figure 2:**
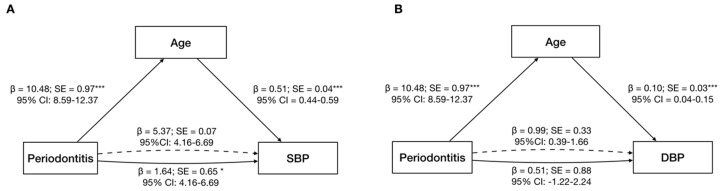
Mediation analysis of age for the association between periodontitis and systolic blood pressure (**A**) and diastolic blood pressure (**B**). ß—Standardized estimates; CI—Confidence interval; DBP—Diastolic Blood Pressure; SBP—Systolic Blood Pressure; SE—Standard error. * *p* < 0.05, ** *p* < 0.01, *** *p* < 0.001. Solid lines = direct effect; dashed lines = indirect effect.

A mediation model was constructed to examine the relationship between an independent variable (periodontitis) and dependent variable (SBP and DBP), via the inclusion of a mediator (age). A statistically significant model was observed when age was included as mediator of the association between periodontitis and SBP (Periodontitis → Age, *p* < 0.001, 95% CI 8.59–12.37; Age → SBP, *p* < 0.001, 95% CI 0.44–0.59; and Periodontitis → SBP, *p* < 0.05, 95% CI 4.16–6.69). An indirect effect (76.6%) of periodontitis on SBP mediated by Age was confirmed (β = 5.37, 95%CI 4.16–6.69). Similarly, a statistically significant model was observed when age was included as mediation in the association between periodontitis and DBP (Periodontitis → Age, *p* < 0.001, 95% CI 8.59–12.37; Age → DBP, *p* < 0.001, 95% CI 0.04–0.15; and Periodontitis → DBP, *p* = 0.562, 95% CI −1.22–2.24), with a statistically significant indirect effect (66.0%) of periodontitis on DBP (β = 0.99, 95% CI 0.39–1.66) (Figure 2).

## 4. Discussion

The results of this study confirmed an association between high BP and periodontitis, analyzing periodontal clinical parameters and traditional cardiovascular risk factors in a well-characterized representative adult population in Portugal. These effects on BP were confounded by the age factor as previously highlighted [43,44], and also a major covariate for periodontal status previously reported in this population [28].

Several lines of evidence suggest that periodontitis is associated with an increased risk of high BP [18,22,43,45,46]. Still, our data raise the hypothesis of a possible linear relationship between different measurements of gingival health and BP measurements. Indeed, a linear association between gingival bleeding on probing and SBP and DBP were observed, similarly to previous evidence [18,22,45,46]. In other words, the more severe the periodontitis, the higher the mean BP. Thus, it is possible that long-lasting and persistent local gingival inflammation can efficiently translate into systemic effects [47,48] and negatively impact BP [18,22]. Inconclusive evidence of a positive effect of periodontal treatment on BP has been reported. Some studies had investigated the impact of non-surgical periodontal therapy (NSPT) on BP levels [49,50], and only three randomized controlled trials (RCTs) reported that intensive NSPT led to a reduction in BP levels [43,51,52]. A recent systematic review reported a reduction in BP after NSPT, ranging from 3 to 12.5 mmHg and 0 to 10 mmHg in SBP and DBP, respectively [18].

In addition, our results showed that participants with higher PPD levels had higher mean SBP and DBP levels, while CAL had the inverse impact on BP. In this context, our results extend the previous evidence on which best measure of exposure links periodontitis and systemic health [53,54]. In this study, two measures of active gingival inflammation (BoP and PPD) were correlated with a greater association with BP [53].

In the literature, the frequency of undiagnosed hypertension has been found to range between 16%–50% [2,55,56,57,58]. Indeed, our study depicted 15% of undiagnosed hypertension participants. Besides, among the undiagnosed hypertension participants, 62.5% of them had periodontitis. Undiagnosed hypertension leads to high cardiovascular mortality and mobility events and remains poorly documented [56,59]. Our results may support dental offices as being potential primary care locations for undiagnosed hypertension screening and monitoring.

Our data show that patients with moderate and severe periodontitis are more likely to be diagnosed with hypertension, in line with current evidence [18]. Interestingly, the odds ratio of having high BP were greater for moderate (OR = 2.60, 95% CI: 1.82–3.72) and severe (OR = 2.20, 95% CI: 1.57–3.08) periodontitis, whereas in participants not taking antihypertensive drugs, only moderate cases were significantly associated with a higher odds ratio of high BP (OR = 2.60, 95% CI: 1.61–4.21).

A large epidemiological study on 102,330 adult participants from the Parisian medical centers assessed whether oral health conditions were linked with the risk of hypertension [60]. While individuals who were 65 or older had no association between oral variables and the risk of hypertension, masticatory function, poor oral hygiene, and oral inflammation were associated with a higher risk of hypertension for those under 65 years old. Similarly, age was a key factor in our study confirmed via mediation analysis, and we also corroborated the associations of hypertension with worse periodontal status following a linear relationship.

The possible biological mechanisms linking periodontitis and high BP point towards the systemic inflammation status related to periodontitis and the spread of oral microbiota into the bloodstream that mediates vascular dysfunction through bacteremia [61,62]. Additionally, T and B lymphocytes, as well as monocytes/macrophages, are primed in an inflamed periodontium increasing the risk for vascular dysfunction, hypertension and atherosclerosis [63,64]. Furthermore, Porphyromonas gingivalis has been recently loomed to trigger an immune reaction, enhancing the sensitivity to pro-hypertensive factors such as low-dose angiotensin II [25]. Importantly, it has been proven that treating severe periodontitis reduces systemic inflammation levels and improves endothelial function [43,65,66].

The results provided by our investigation have some notable strengths, but also limitations. This report is based on a cross-sectional survey that precludes any deduction of causality or temporal relationship between the analyzed variables, notwithstanding reverse causality whereby high BP leads to periodontal inflammation and cannot be excluded as previously stated [18]. A further limitation is the fact that our analyses were based on a single BP measure, a method that has its inherent and widely accepted bias [34]. Nevertheless, our approach of using a single measure of BP might represent a feasible and “real world” method of screening patients for hypertension within a busy dental setting. Dentists and other oral health professionals could represent an underestimated source of support and opportunity to help relieve the burden of hypertension. Additional strengths in our study included the detailed full-mouth protocol of dental examination so as to increase accuracy and precision [67], and a comprehensive analysis plan.

Further longitudinal studies are needed to establish the nature of the association between periodontitis and BP, and in particular raise the possibility that periodontal therapy could be a novel non-pharmacological intervention to alleviate hypertension burden and complications.

## 5. Conclusions

Our data highlight that patients with periodontitis, particularly those with higher levels of gingival inflammation and deep periodontal pockets, have the highest risk for having both SBP and DBP. Moreover, smoking habits, gender, age and BMI were independently associated with raised BP. These results suggest that periodontitis may have an important negative impact on BP.

## Figures and Tables

**Table 1:** Baseline characteristics of participants according to periodontal status (*n* = 1057).

	No Periodontitis (*n* = 423)	Periodontitis (*n* = 634)	*p*-Value ^#^
Age, mean (SD)	54.8 (17.8)	65.2 (13.5)	<0.001
Gender, *n* (%)			
Female (*n* = 610)	283 (46.4)	327 (53.6)	<0.001
Male (*n* = 447)	140 (31.3)	307 (68.7)	
Race, *n* (%)			
Caucasian (*n* = 916)	359 (39.2)	557 (60.8)	0.164
Black (*n* = 130)	57 (43.8)	73 (56.2)	
Asian (*n* = 11)	7 (63.6)	4 (36.4)	
Education Level, *n* (%)			
No education (*n* = 42)	11 (26.2)	31 (73.8)	<0.001
Basic (*n* = 410)	134 (32.7)	276 (67.3)	
Medium (*n* = 490)	205 (41.8)	285 (58.2)	
Higher (*n* = 115)	73 (63.5)	42 (36.5)	
Smoking Habits, *n* (%)			
Never (*n* = 624)	294 (47.1)	330 (52.9)	<0.001
Former (*n* = 288)	83 (28.8)	205 (71.2)	
Current (*n* = 145)	46 (31.7)	99 (68.3)	
Income, mean (SD) (€)	1097.0 (767.3)	981.5 (667.9)	0.023
Clinical Variables			
Hypertension, *n* (%)			
No (*n* = 357)	188 (52.7)	169 (47.3)	<0.001
Yes (*n* = 700)	235 (33.6)	465 (66.4)	
SBP, mean (SD)	129.5 (20.1)	136.5 (20.4)	<0.001
DBP, mean (SD)	77.9 (13.2)	79.6 (13.6)	0.082
SBP ≥ 140 mmHg, *n* (%)			
No (*n* = 681)	308 (45.2)	373 (54.8)	<0.001
Yes (*n* = 376)	115 (30.6)	261 (69.4)	
Taking Antihypertensive Medication, *n* (%)			
Yes (*n* = 532)	172 (32.3)	360 (77.7)	<0.001
No (*n* = 525)	251 (47.8)	274 (52.2)	
Number of medical conditions, mean (SD)	1.89 (1.6)	2.36 (1.53)	<0.001
Diabetes Mellitus, *n* (%)			
Yes (*n* = 204)	53 (26.0)	151 (74.0)	<0.001
No (*n* = 853)	370 (43.4)	483 (56.6)	
BMI, mean (SD)	27.1 (4.9)	27.5 (4.7)	0.051
Periodontal Clinical Parameters, Mean (SD)			
Missing Teeth (*n*)	6.6 (6.1)	10.8 (7.1)	<0.001
Mean PPD (mm)	1.51 (0.30)	2.22 (0.83)	<0.001
PPD ≥ 3 mm (%)	7.2 (8.1)	29.9 (24.0)	<0.001
PPD ≥ 4 mm (%)	6.1 (16.0)	13.0 (17.8)	<0.001
PPD ≥ 5 mm (%)	1.1 (4.5)	8.8 (15.1)	<0.001
PPD ≥ 6 mm (%)	0.1 (0.2)	2.7 (6.7)	<0.001
PPD ≥ 7 mm (%)	0.0 (0.1)	1.3 (4.2)	<0.001
Mean CAL (mm)	1.72 (0.35)	3.39 (1.52)	<0.001
CAL ≥ 3 mm (%)	15.2 (12.3)	56.6 (26.0)	<0.001
CAL ≥ 4 mm (%)	3.9 (5.3)	37.8 (27.9)	<0.001
CAL ≥ 5 mm (%)	0.8 (2.0)	25.2 (26.0)	<0.001
CAL ≥ 6 mm (%)	0.3 (1.1)	15.2 (21.9)	<0.001
CAL ≥ 7 mm (%)	0.1 (0.7)	9.0 (16.9)	<0.001
Mean Rec (mm)	0.22 (0.27)	1.18 (1.15)	<0.001
PISA (mm^2^)	12.2 (26.3)	65.9 (137.1)	<0.001
PESA (mm^2^)	177.7 (76.8)	218.8 (170.8)	0.002
PI (%)	12.3 (21.1)	30.6 (33.2)	<0.001
BoP (%)	5.6 (9.3)	20.9 (23.6)	<0.001

^#^ Chi-square test for categorical variables, Mann–Whitney test for continuous variables. BMI—Body Mass Index; BoP—Bleeding on Probing; CAL—Clinical Attachment Level; DBP—Diastolic Blood Pressure; PESA—Periodontal Epithelial Surface Area; PI—Plaque Index; PPD—Probing Pocket Depth; Rec—gingival recession; SBP—Systolic Blood Pressure; SD—Standard Deviation.

**Table 2:** Odds ratios (OR) and correspondent 95% confidence intervals (CI) towards hypertension, according to the periodontal status, calculated within binary logistic regression analyses for different adjustment levels.

	Periodontitis OR (95%CI)	Stage 1 (Mild) OR (95%CI)	Stage 2 (Moderate) OR (95%CI)	Stage 3 (Severe) OR (95%CI)
**All Participants (*n* = 1057)**				
Model 1	2.20 (1.70–2.86) ***	1.72 (1.10–2.57) **	2.60 (1.82–3.72) ***	2.20 (1.57–3.08) ***
Model 2	2.14 (1.64–2.78) ***	1.69 (1.13–2.52) *	2.54 (1.77–3.63) ***	2.12 (1.51–2.98) ***
Model 3	2.36 (1.64–2.78) ***	1.79 (1.19–2.70) **	2.87 (1.97–4.17) ***	2.36 (1.66–3.36) ***
Model 4	2.31 (1.75–3.04) ***	1.75 (1.15–2.66) **	2.78 (1.90–4.01) ***	2.32 (1.62–3.32) ***
Model 5	1.24 (0.90–1.71)	1.36 (0.84–2.18)	1.41 (0.92–2.15)	1.04 (0.69–1.55)
Participants Not Taking Antihypertensive Medication (*n* = 525)				
Model 1	1.85 (1.27–2.70) ***	1.69 (0.96–2.95)	2.60 (1.61–4.21) ***	1.36 (0.82–2.70)
Model 2	1.81 (1.24–2.65) **	1.66 (0.95–2.90)	2.55 (1.57–4,14) ***	1.32 (0.79–2.22)
Model 3	1.89 (1.28–2.79) ***	1.66 (0.94–2.91)	2.67 (1.64–3.36) ***	1.44 (0.85– 2.44)
Model 4	1.86 (1.26–2.75) **	1.66 (0.94–2.92)	2.60 (1.59–4.26) ***	1.43 (0.85–2.42)
Model 5	1.24 (0.81–1.90)	1.36 (0.75–2.45)	1.62 (0.96–2.75)	0.82 (0.47–1.46)

Model 1—Unadjusted model; Model 2—Includes adjustment for gender; Model 3—Includes adjustment for gender and smoking habits; Model 4—Includes adjustment for gender, smoking habits and BMI; Model 5—Includes adjustment for gender, smoking habits, BMI and age. Statistically significant: * *p* < 0.05; ** *p* < 0.01; *** *p* < 0.001.

**Table 3:** Multiple unadjusted linear regression models for all participants and according to the use of antihypertensive medication towards Systolic Blood Pressure (SBP) and Diastolic Blood Pressure (DBP) readings.

	All Participants (*n* = 1057)		No Antihypertensive Use (*n* = 525)		Antihypertensive Use (*n* = 532)	
	ß Coefficient (SE)	*p*	ß Coefficient (SE)	*p*	ß Coefficient (SE)	*p*
SBP (mmHg)						
Age (years)	0.44 (0.04)	<0.001	0.44 (0.04)	<0.001	0.40 (0.09)	<0.001
BMI (kg/m^2^)	0.52 (0.12)	<0.001	0.40 (0.17)	0.018	0.54 (0.18)	0.003
%PPD ≥ 6 mm	45.47 (12.37)	<0.001	-	-	-	-
PESA (Total)	−0.02 (0.01)	0.001	-	-	-	-
BoP (%)	7.86 (3.14)	0.012	7.42 (3.75)	0.048	-	-
%PPD ≥ 7 mm	-	-	-	-	78.36 (0.18)	<0.001
Missing teeth (*n*)	-	-	-	-	0.28 (0.13)	0.031
DBP (mmHg)						
Age (years)	0.07 (0.03)	0.009	0.09 (0.04)	0.009	-	-
BMI (kg/m^2^)	0.33 (0.09)	<0.001	0.42 (0.13)	0.002	-	-
%PPD ≥ 6 mm	40.73 (9.93)	<0.001	-	-	22.97 (9.76)	0.019
PESA (Total)	−0.01 (0.00)	0.030	-	-	-	-
BoP (%)	10.10 (2.44)	<0.001	-	-	10.88 (2.97)	<0.001
Mean CAL (mm)	−0.79 (0.39)	0.045	-	-	-	-

BMI—Body Mass Index; BoP—Bleeding on Probing; CAL—Clinical Attachment Level; DBP—Diastolic Blood Pressure; PESA—Periodontal Epithelial Surface Area; PPD—Periodontal Pocket Depth; SBP—Systolic Blood Pressure.

## Supplementary Materials

The following are available online at https://www.mdpi.com/2077-0383/9/5/1585/s1, Figure S1: ROC Analysis using periodontal diagnosis, Figure S2: ROC Analysis using periodontal measures, Table S1: Comparison of baseline characteristics between healthy, gingivitis and periodontitis stages (N = 1057).
